# Ultrasound-guided ethanol sclerotherapy versus laparoscopic surgery for endometriomas: a randomized clinical trial in a real-world setting

**DOI:** 10.1007/s00404-025-08205-1

**Published:** 2025-11-04

**Authors:** Amparo García-Tejedor, Rodrigo Guevara-Peralta, Jose Manuel Martinez-Garcia, Shiana Corbalán, Mauricio Agüero, Maria Gomez-Romero, Marta Cararach, Marta Castellarnau, Mariví Rodríguez, Ana Cristina Lou-Mercadé, Laura Costa, Maria Jose Rodríguez, Eva Huguet, Manuel Carreras, Ana Belen Castel-Segui, Maria Font-Roig, Susana Royo, Nuria Sarasa, Beatriz Candas, Samuel Perez-Carton, Carlos Ortega, Maria Jesus Pla, Jordi Ponce

**Affiliations:** 1 Department of Gynecology, Facultat de Medicina, Hospital Universitari Bellvitge, Universitat de Barcelona, , Hospitalet de Llobregat, Bellvitge Hospital, Ave. Feixa Llarga, Sn., 08970 Barcelona, Spain; 2 IDIBELL, Instituto de Investigación Biomédica de Bellvitge, Hospitalet de Llobregat, Barcelona, Spain; 3 Facultat de Medicina, Departament de Ciències Clíniques, Universitat de Barcelona, Hospitalet de Llobregat, Barcelona, Spain; 4 Department of Gynecology, Hospital Los Arcos, Murcia, Spain; 5 Department of Gynecology, Hospital del Mar, Barcelona, Spain; 6 Department of Gynecology, Hospital Joan XXIII, Tarragona, Spain; 7 Department of Gynecology, Hospital de Viladecans, Barcelona, Spain; 8 Department of Gynecology, Consorci Integral, Hospitalet de Llobregat, Barcelona, Spain; 9 Department of Gynecology, Hospital San Pedro, Logroño, Spain; 10 Department of Gynecology, Hospital Clínico Universitario Lozano Blesa, Saragossa, Spain; 11 Department of Gynecology, Parc Taulí, Sabadell, Bacelona, Spain; 12 Department of Gynecology, Hospital Universitario Central de Asturias, Asturias, Spain; 13 Department of Gynecology, Hospital Universitari Mutua de Terrasa, Barcelona, Spain; 14 Department of Gynecology, Hospital de Sant Boi, Barcelona, Spain; 15 Department of Gynecology, Hospital Son Espases, Mallorca, Spain; 16 Department of Gynecology, Hospital Santa Caterina, Girona, Spain; 17 Department of Gynecology, Hospital General, Valencia, Spain; 18 Department of Gynecology, Hospital de Granollers, Barcelona, Spain; 19 Department of Biochemistry and Molecular Biology, Clinical Laboratory, Hospital Universitari Bellvitge, Hospitalet de Llobregat, Barcelona, Spain

**Keywords:** Endometrioma, Infertility, Cystectomy, Sclerotherapy, Recurrences, Pain, Ultrasound-guided aspiration, Surgery

## Abstract

**Purpose:**

To compare the efficacy of ultrasound-guided alcohol sclerotherapy versus laparoscopic cystectomy for the management of ovarian endometriomas, focusing on complications, recurrence, pain relief, and healthcare costs.

**Methods:**

We conducted a multicentre, randomized clinical trial across 20 centers in Spain. A total of 167 women aged 18–40 years with ovarian endometriomas (35–100 mm) were recruited between June 2018 and June 2022. Participants were randomized to receive either ultrasound-guided aspiration with ethanol sclerotherapy or standard laparoscopic cystectomy. Complications were graded using the Clavien–Dindo classification. Pain was assessed using a visual analogue scale (VAS) before and six months after treatment. Recurrence was defined as the reappearance of a cystic lesion at the treated site and analyzed using Kaplan–Meier curves and log-rank tests. The primary analysis followed an *intention-to-treat* approach and included 158 patients (sclerotherapy: *n* = 84; cystectomy: *n* = 74). The *per-protocol* analysis included 92 patients (sclerotherapy: *n* = 57; cystectomy: *n* = 37). Direct hospital costs, complication rates, recurrence, and pain relief were compared between groups.

**Results:**

Intention-to-treat analyses show that complications were low in both groups (12%), most of which were Grade I–II, although 4.1% were Grade III in the surgery group. The cost of sclerotherapy was significantly lower (€472 vs. €2128, *p* < 0.001). In per-protocol analyses, the cyst recurrence or reappearance was similar between the two groups, with rates of 25.7% (9 of 35) in the surgery group and 22.8% (13 of 57) in the sclerotherapy group (*p* = 0.16). Pain was improved or completely resolved in 49 of 55 cases (89.1%) in the sclerotherapy group and in 21 of 32 cases (65.7%) in the laparoscopic surgery group (*p* = 0.05).

**Conclusions:**

Ultrasound-guided alcohol sclerotherapy is a safe, cost-effective alternative to laparoscopic cystectomy for the treatment of endometriomas, with comparable recurrence rates and pain relief. Clinical Trial Registration: https://clinicaltrials.gov/search?term=NCT03571776. Registered May 5, 2018.

## What does this study add to the clinical work

**Table:** 

1. Ultrasound-guided alcohol sclerotherapy demonstrates comparable safety and recurrence rates to laparoscopic cystectomy, with a trend toward superior pain relief and significantly reduced hospital costs. 2. These findings support sclerotherapy as a cost-effective and minimally invasive alternative for the management of ovarian endometriomas in women of reproductive age.	

## Introduction

Ovarian endometrioma affects approximately 6.1% of women of reproductive age [1] and is associated with chronic pain, infertility, and reduced quality of life [2]. Surgical intervention has traditionally been the mainstay of treatment, but concerns over postoperative complications and fertility preservation have driven interest in alternative approaches [3–5].

Clinical guidelines recommend hormonal therapies, including combined oral contraceptives and progestogens, for symptom management [6]. Laparoscopic surgery remains a widely accepted option for endometriosis-related pain [7], utilizing excision, diathermy, or ablation. However, the risk of adhesions and complications, particularly in cases of recurrent disease, remains a concern. While major complications after laparoscopic surgery are reported at 0.46% [8], endometriosis-related adhesions may increase these risks.

Minimally invasive alternatives, such as ultrasound (US)-guided aspiration and sclerotherapy, are emerging as promising treatments for ovarian cysts, including endometriomas. These techniques have shown recurrence rates of 0–62.5% over 12–24 months [9–18], comparable to laparoscopic rates of 15–30% [19, 20], while potentially preserving ovarian function [16, 21].

This study aims to compare the efficacy of ultrasound-guided ethanol sclerotherapy versus laparoscopic surgery for the treatment of endometriomas in a real-world clinical setting. To assess the risk–benefit profile of the procedure, the primary outcomes were the incidence of complications and the cyst recurrence or reappearance rates. Secondary outcomes include the evaluation of pain relief, a basic cost analysis, and the identification of key risk factors associated with recurrence to inform and optimize patient management strategies.

## Methods

This prospective, randomized, open-label, multicenter phase III trial evaluated the efficacy of ultrasound (US)-guided ethanol sclerotherapy versus laparoscopic surgery for the treatment of ovarian endometriomas in a real-world setting. The study was conducted between June 5, 2018, and June 30, 2022, in accordance with the protocol approved by the local ethics committee (PR031/18) and registered on ClinicalTrials.gov (NCT03571776). For the purposes of this manuscript, both an intention-to-treat (ITT) and a per-protocol (PP) analysis were performed. The trial adhered to the ethical principles outlined in the Declaration of Helsinki and was funded by the Carlos III Health Institute (grant number PI16/00801).

**Fig. 1:**
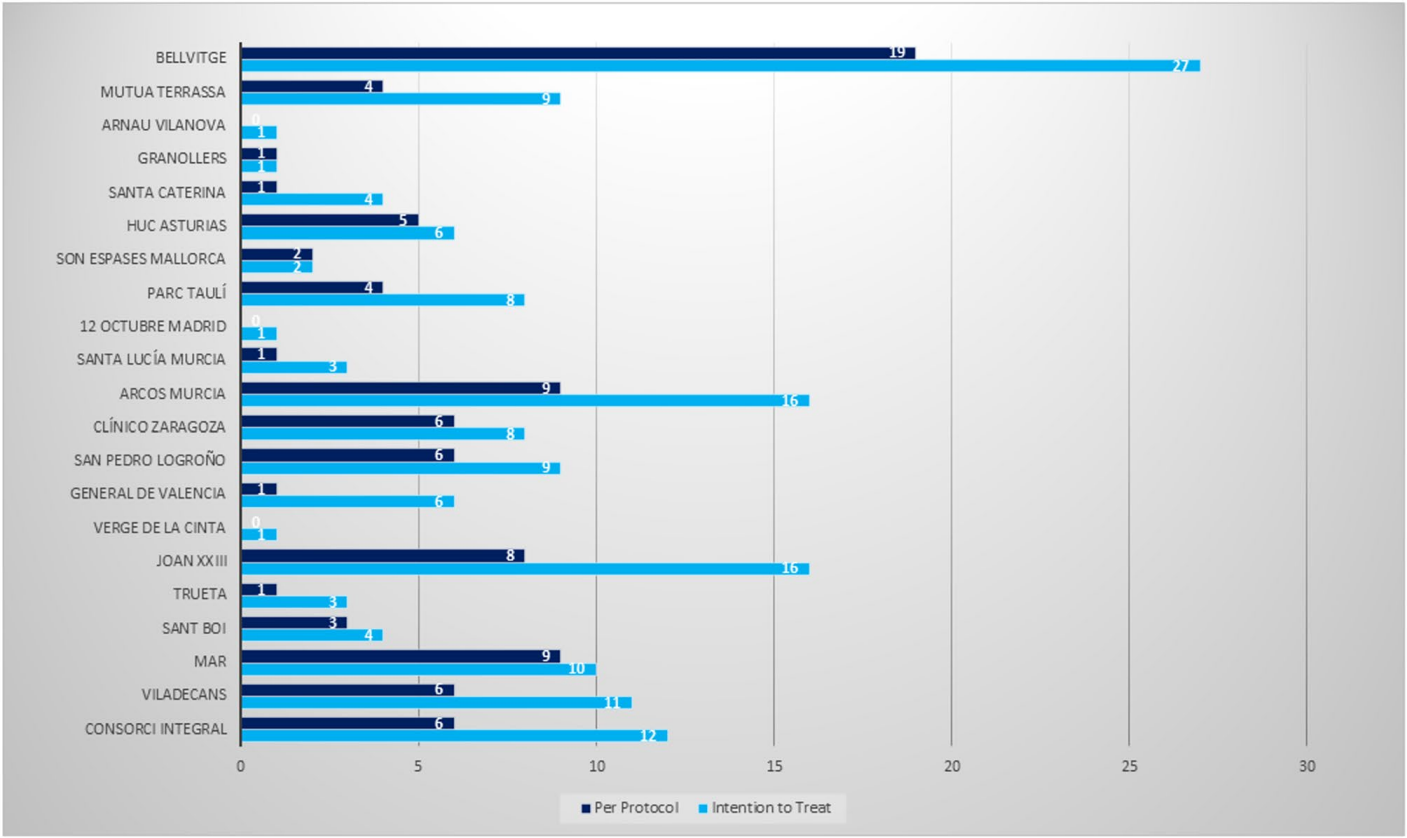
Patients Included per hospital

A total of 167 patients with suspected ovarian endometrioma were recruited from 20 secondary and tertiary endometriosis referral centres across Spain (Fig. 1 shows the number of patients enrolled by each hospital where they were treated to provide real-world data). Eligible participants were women aged 18–45 years with US features predictive of endometrioma according to the International Ovarian Tumor Analysis (IOTA) criteria, measuring 35–100 mm if symptomatic or 50–100 mm if asymptomatic, with cyst persistence for ≥ 3 months. Exclusion criteria included abnormal coagulation, history of gynecologic cancer, pregnancy, menopause, or severe extraovarian endometriosis confirmed by MRI (no data were collected from this subgroup). All patients signed an informed consent form.

Participants were randomized (1:1) to ultrasound-guided aspiration with ethanol sclerotherapy or laparoscopic cystectomy using stratified block randomization to balance age (≤ 32 vs > 32 years), history of ovarian surgery (yes vs no), and recruiting center (20 hospitals). This yielded 80 strata, each randomized in fixed blocks of six via a centralized, web-based system with concealed allocation. After eligibility was confirmed online by the local investigator, treatment allocation was revealed through the platform. The assigned procedure was performed or supervised by the local investigator. Given the intrinsic differences between surgical and non-surgical arms, some patients withdrew after randomization. To assess the specific effects of each intervention, a per-protocol analysis excluded crossovers and cases in which the allocated procedure could not be completed due to technical limitations.

### Procedures

**Fig. 2:**
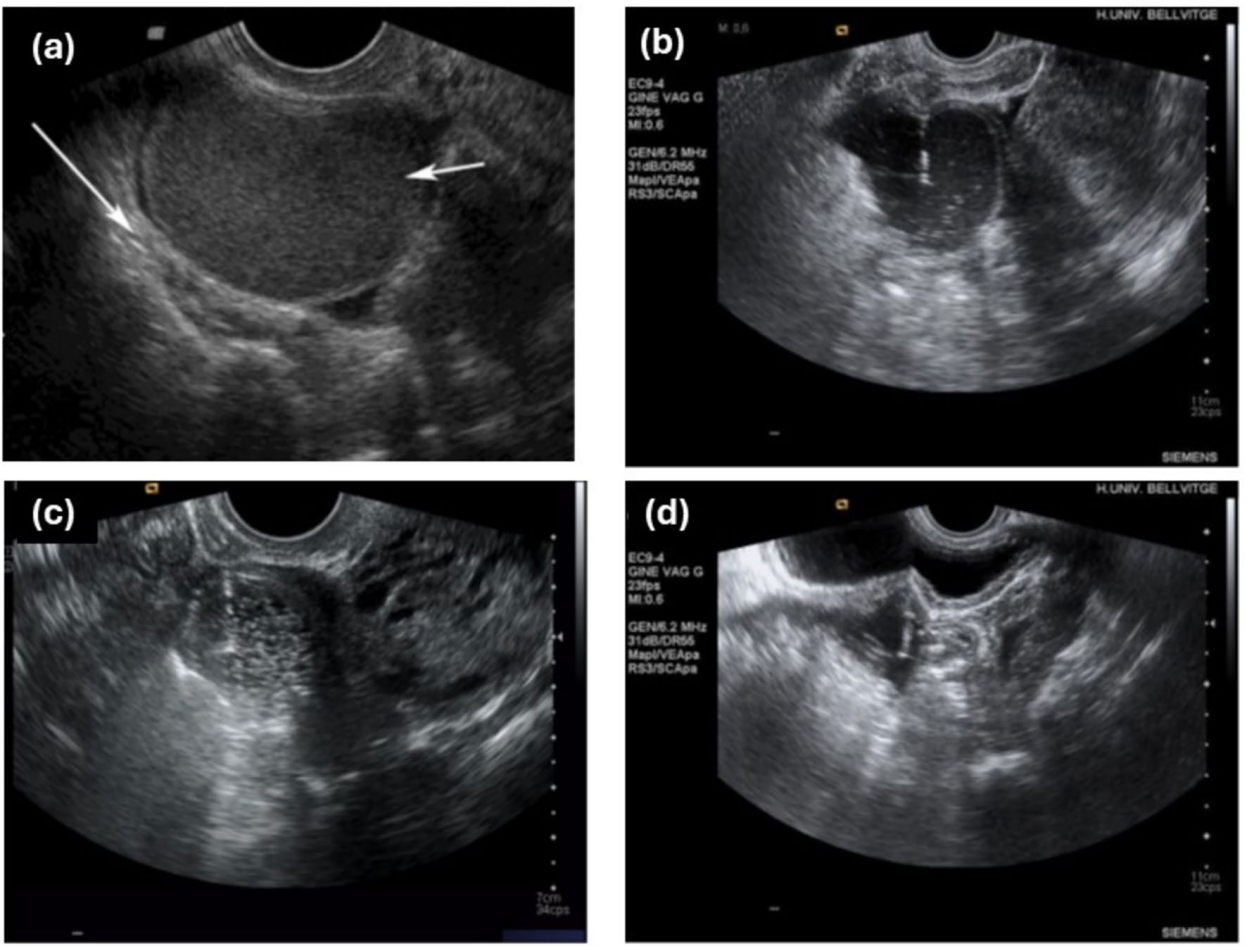
Ethanol sclerotherapy procedure. (a) Diagnosis of stable endometrioma (3-month follow-up) between 30–100 mm in diameter. (b) US-guided cyst aspiration and saline lavage. (c) Ethanol intracyst infusion and sclerosis (2/3 of the aspirated volume, maximum 100cc of ethanol). (d) Complete aspiration of the cyst

*Ultrasound-guided aspiration and ethanol sclerotherapy (US-ES)*: Procedures were performed in an outpatient setting under ultrasound guidance, preferably transvaginal or transabdominal in virginal patients or when vascular structures were identified along the puncture tract [22, 23]. After vaginal disinfection with povidone, a 17-gauge spinal follicular needle (BD Medical, Franklin Lakes, NJ, USA) was introduced into the cyst cavity for complete aspiration. When fluid was dense, saline dilution was applied, never exceeding the aspirated volume. Multiple saline washes were performed, leaving a small residual volume to prevent leakage. Sterile 100% ethanol (Xalabarder Pharmacy, Barcelona, Spain) was instilled at two-thirds of the aspirated volume (maximum 100 mL), maintained for 15 min, and then completely evacuated, followed by a final saline rinse (Fig. 2). Aspirated content was submitted for cytology. Patients received sublingual diazepam (30 min before) and oral ibuprofen (60 min before) but no anesthesia, sedation, or prophylactic antibiotics.

*Laparoscopic cystectomy*: All surgeries were performed under general anesthesia by gynecological surgeons specialized in minimally invasive surgery and endometriosis management. After adhesiolysis and ovarian mobilization when required, the cyst was opened at the antimesenteric border with monopolar cautery, aspirated, and the pseudocapsule removed by traction–countertraction with two 5 mm forceps (“stripping technique”). Hemostasis was achieved with bipolar coagulation when necessary. Surgical procedures followed international recommendations to minimize damage to healthy ovarian tissue, with intraoperative variations adapted to disease extent and adhesions.

### Variables and outcomes

Baseline variables included age, body mass index, history of previous endometriosis surgery, infertility, pain (visual analog scale, VAS), transvaginal ultrasound, antral follicle count (AFC), Anti-Müllerian Hormone (AMH), CA125 and HE4 levels, and MRI in cases of suspected deep infiltrating endometriosis. All procedures were performed by experienced gynecologists following standardized protocols. Follow-up consisted of US every 6 months during the first year and annually thereafter for a minimum of 2 years, assessing recurrence and symptom relief.

Primary outcomes were complication rates (classified according to Clavien–Dindo [24]) and recurrence (defined as the presence of an adnexal cyst ≥ 30 mm in the treated ovary during follow-up). Secondary outcomes included pain relief (VAS before and 6 months after the procedure) and direct hospital costs. Cost analysis, based on data from the financial system of Bellvitge University Hospital, included operating room use, staff salaries, supplies, medications, inpatient services, complications, and length of stay (Table 1). Although not designed for cost-effectiveness evaluation, a basic cost analysis was performed to estimate the economic burden of the procedures. All costs were expressed in euros.

**Table 1:** Estimation of direct medical cost of patients in both groups (euros)

	Surgery	Sclerosis	Differences
Staff labour	536,90	105,83	431
Operation room occupation	459,73	0	460
Consumables materials	350,43	70,46	280
Medication	67,09	2,99	64
Ultrasound amortization	0	12,3	-12
Inpatient costs per day	362,55	0	363
Miscellaneous costs	412,41	74,66	338
Total in hospital direct costs	**2.189,10**	**256,97**	**1.923**

### Statistical analysis

Sample size was initially calculated using AMH as the primary endpoint, but pilot data (*n* = 40) showed that AMH levels were mainly associated with baseline values [10]. Recurrence rates of 15% after surgery and 5% after sclerotherapy suggested a need for 140 patients per group; however, given the higher rate of severe complications in the surgical arm, the primary endpoint was redefined to complication rates. Based on pilot data (14% vs. 3.3%), the recalculated sample size using GRANMO (*α* = 0.05, *β* = 0.2, 1:1 allocation) was 107 patients per group. Complication rates, as the main outcome, were compared using the chi-square test, while recurrence was analyzed with Kaplan–Meier curves and log-rank tests. Risk factors for recurrence were evaluated with Cox proportional hazards models, applying bootstrapping (1000 iterations) to identify the optimal cyst diameter threshold, and hospital costs were compared using Student’s t-test. An interim analysis at midpoint enrollment assessed safety and recurrence; although no major safety concerns were observed, recruitment decline and the low probability of reaching the target sample size led to early termination of the study for futility. Statistical significance was set at *p* < 0.05, and analyses were performed with R software (version 3.5.0).

## Results

**Fig. 3:**
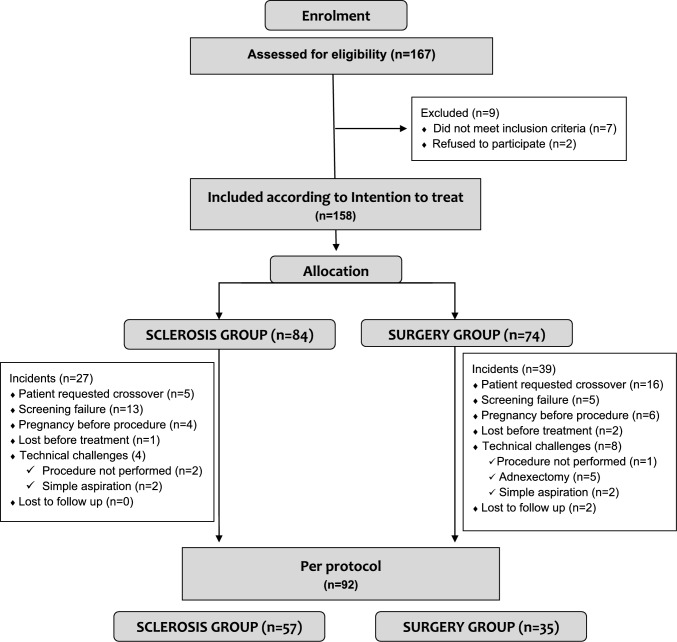
Flow Chart Diagram

A total of 167 patients were recruited, of whom 158 were included in the intention-to-treat analysis (Fig. 3). Crossover occurred in 16 of 74 patients (21.6%) allocated to surgery, who ultimately underwent sclerotherapy, compared with 5 of 84 patients (6.0%) in the sclerotherapy group who opted for surgery (*p* < 0.01). This reflects a significantly greater preference for the less invasive approach. The per-protocol population comprised 92 patients (sclerotherapy: *n* = 57; cystectomy: *n* = 37). Table 2 provides a comprehensive summary of the clinical characteristics and cyst features for the ITT and PP approach.

**Table 2:** Baseline Patient Characteristics

	**Intention-to-Treat Cohort**			**Per Protocol Cohort**		
	**Sclerotherapy** **(n = 84)**	**Surgery** **(= 74)**	**p**	**Sclerotherapy (n = 57)**	**Surgery** **(= 35)**	**p**
Age (years) mean (SD)	34 (6.4)	33 (6.4)	0.29	35 (5.7)	32 (5.9)	0.046
BMI (kg/m^2)^ mean (SD)	24.5 (4.6)	24.2 (6.7)	0.77	24.5 (4.4)	23.9 (6.7)	0.71
AMH (ng/mL) mean (SD)	1.9 (2.1)	2.4 (1.9)	0.18	1.9 (1.9)	2.7 (2.1)	0.09
Ca125 (U/mL) mean (SD)	75.2 (97)	74.8 (77.5)	0.62	81.6 (104)	95 (92.6)	0.63
Infertility No Yes			0.12			0.13
	43 (57.3%) 32 (42.7%)	43 (71.7%) 17 (34.7%)		32 (56.1%) 25 (43.9%)	26 (74.3%) 9 (25.7%)	
Previous ovarian surgery for endometriosis, n (%) No Yes	69 (82.1%) 15 (17.9%)	60 (81.1%) 14 (18.9%)	0.86	50 (87.7%) 7 (12.3%)	33 (94.3%) 2 (5.7%)	0.26
Pain at diagnosis	7 (9.2%) 69 (90.8%)		0.68			0.76
None		8 (12.9%)		9 (7.3%)	4 (3.1%)	
Yes		54 (87.1%)		48 (92.3%)	31 (96.9%)	
Hormonal therapy, n (%) No Yes			0.82			1
	41 (54.7%) 34 (45.3%)	33 (54.1%) 28 (65.9%)		33 (57.9%) 24 (42.1%)	20 (55.9%) 15 (44.1%)	
**Endometrioma features**						
Median size by US (Q1;Q3), mm	60 (50;73)	63 (51;78)	0.12	60 (50;73)	65 (56–79)	0.054
Localization Bilateral Unilateral			0.14			0.48
	15 (20.3%) 59 (79.7%)	6 (9.7%) 56 (90.3%)		11 (19.3%) 46 (80.7%)	4 (11.4%) 31 (88.6%)	
Number of cyst locules Unilocular Bilocular	61 (84.7%) 11 (15.3%)	45 (76.3%) 14 (23.7%)	0.32	50 (87.3%) 7 (12.7%)	24 (69.6%) 11 (31.4%)	0.058
AFC, mean (SD)	9.4 (7.3)	9.8 (6.9)	0.72	9.4 (7.1)	11.2 (6.3)	0.21

AFC = antral follicle count; BMI = body mass index; SD = standard deviation; US = ultrasound

### Intention-to-treat approach

Procedural success was achieved more frequently with ethanol sclerotherapy than with surgery (61/84, 72.6% vs. 38/74, 51.4%; *p* < 0.009) (Table 3). In the sclerotherapy group, technical difficulties occurred in 5/84 cases (6%)—including painful ethanol extravasation (*n* = 2), dense intracystic fluid (*n* = 2), and a screening error with a non-endometrioma cyst (*n* = 1). As a result, the procedure was successfully completed in 61/66 women (92.5%) in whom it was attempted, with pain managed effectively with oral analgesics. In the surgical group, laparoscopic cystectomy was completed as planned in 38/46 patients (82.6%), although 8 procedures (17.4%) were complicated by severe adhesions requiring alternative approaches (five adnexectomies, two capsule drainages with coagulation, and one bilateral salpingectomy). Extraovarian endometriosis was detected in 31/46 patients (67.4%), mainly peritoneal, and was treated with ablation or electrocoagulation.

**Table 3:** Outcomes Related to the Procedure: Intention-to-Treat Analysis

	**Sclerotherapy** **(n = 84)**	**Surgery** **(n = 74)**	**p value**
Procedure Successfully completed Not possible/change technique^(a)^ Cross-over^(b)^ Not done			0.009
	61 (72.6%) 5 (6%) 9 (10.7%) 9 (10.7%)	38 (51.4%) 8 (10.8%) 8 (10.8%) 20 (27%)	
Admission days, n (%) 0 1 2 3–4			< 0.001
	66 (88%) 9 (12%) - -	15 (18.2%) 19 (35.2%) 13 (24.1%) 7 (13%)	
Complications (Clavien-Dindo), n (%) None Grade I Grade II Grade III^(c)^ Grade IV-V			0.7
	74 (88.1%) 8 (9.5%) 1 (1.2%) 1 (1.2%) –	65 (87.8%) 5 (6.8%) 1 (1.4%) 3 (4.1%) –	
Costs (mean, DS), euros	472 (598)	2128 (709)	< 0.001

^(a)^ Technical difficulties were encountered in 5 cases (6%) in the sclerotherapy group, 2 cases were due to the high density of the fluid, 2 cases were specifically related to painful ethanol extravasation; and one case was due to a screening failure involving a non-endometrioma cyst. In the surgery group, 8 cases (10.8jknjh%) presented challenges, mainly due to extensive pelvic adhesions that complicated dissection

^(b)^ In addition to the five patients in the sclerotherapy group who requested crossover, four additional patients were reassigned due to screening failures: elevated tumour markers (n = 2), endometrioma rupture (n = 3), and increased size and symptoms compared to the previous control in bilateral endometrioma (n = 1). In the surgery group, of the 16 patients who wanted to change, only eight underwent sclerotherapy and the other eight were monitored

^(c)^ Clavien-Dindo Grade III were in the surgery group, one pelvic infection, one haemostasis failure and one due to multiple adhesions; and in the sclerosis group, one pelvic infection in an endometrioma too dense for evacuation and where prophylactic antibiotic was omitted

- gray highlighting shows the statistically significant factors in the univariate analyses

Sclerotherapy was performed as an outpatient procedure in all but one case, where general anesthesia was administered at the patient’s request. Eight additional hospitalizations corresponded to patients who crossed over to surgery. Laparoscopic cystectomy was performed as a major outpatient or one-day surgery in 34/55 cases (61.8%), while 21 patients (38.2%) required a 2–4-day stay, mainly due to complex procedures in the context of extensive adhesions or extra-pelvic endometriosis, which demanded advanced surgical techniques and prolonged recovery. Only one patient required extended hospitalization for postoperative dizziness.

Overall, both interventions were well tolerated, reflecting their minimally invasive profile. Four Clavien–Dindo grade III complications were recorded: three in the laparoscopic group (4.1%; pelvic infection, hemostasis failure, and severe adhesions) and one in the sclerotherapy group (1.1%; pelvic infection following an aborted procedure due to dense cyst content, likely related to omission of antibiotic prophylaxis). Direct hospital costs were significantly lower with sclerotherapy compared to surgery (mean €472, SD 598 vs. €2128, SD 709).

### Recurrence and long-term follow-up (Per protocol approach)

**Fig. 4:**
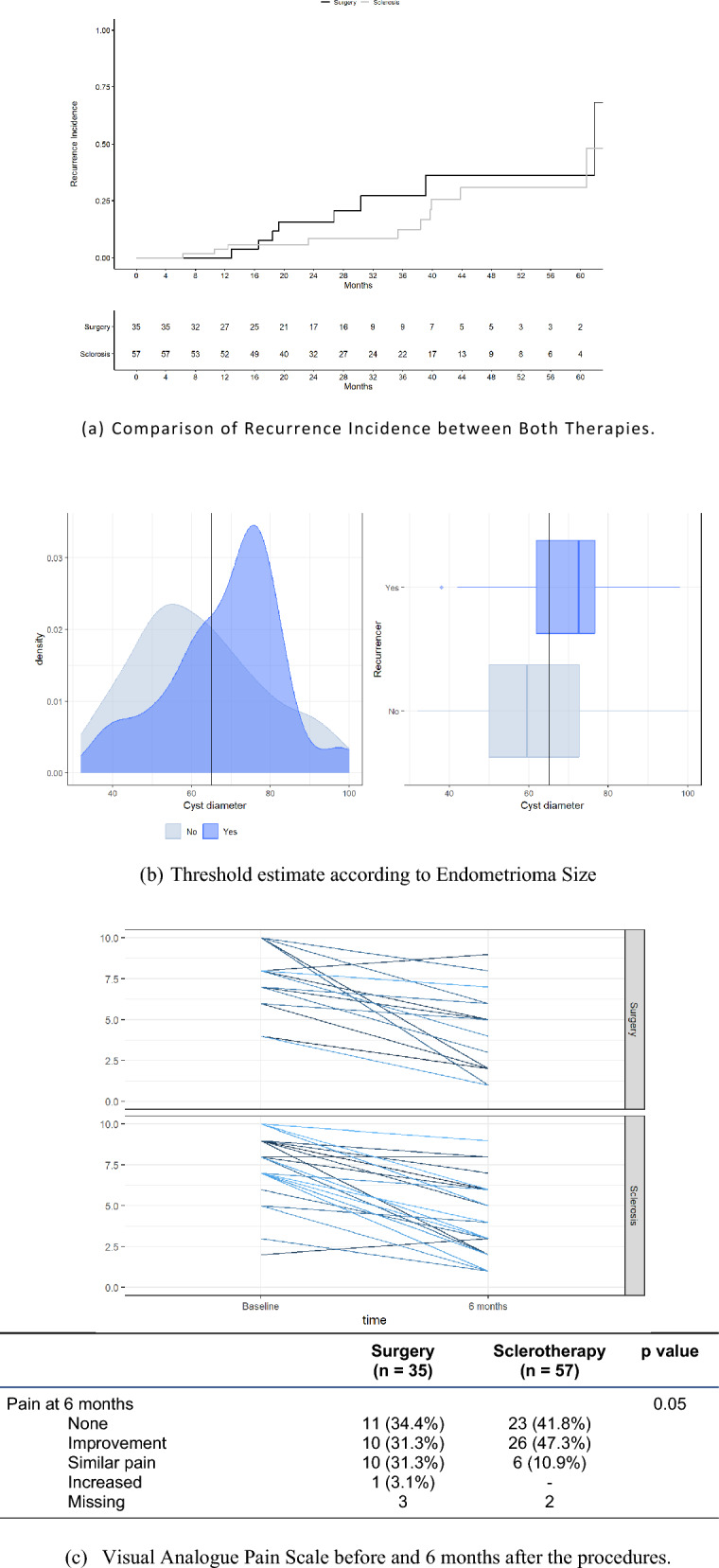
Recurrences and Follow-up. (a) Comparison of Recurrence Incidence between Both Therapies; (b) Threshold estimate according to Endometrioma Size; (c) Visual Analogue Pain Scale before and 6 months after the procedures

After a median follow-up period of 25 months (Q1-Q3 18–36), 22 of 92 cases (23.9%) experienced recurrence. The recurrence rates were comparable between the two groups: 25.7% (9 of 35) in the surgery group and 22.8% (13 of 57) in the sclerotherapy group (*p* = 0.16) (Fig. 4a). Table 4 outlines the variables potentially associated with recurrence. Multivariate analyses did not identify any specific factors associated with recurrence, although older age, BMI and larger cyst size approached statistical significance.

**Table 4:** Risk Factors for Recurrences: Univariate and Multivariate Analysis – Per Protocol

	**No Recurrence** **(n = 70)**	**Recurrence** **(n = 22)**	**p value** **(univariate)**	**HR (95%CI)** **(multivariate)**
Procedure, n (%) Sclerotherapy Surgery			0.16	NS
	44 (77.2%)	13 (22.8%)		
	26 (74.3%)	9 (25.7%)		
Age (years) mean (SD)	33.1 (6.1)	34.8 (5.3)	0.36	1.11 [1.02–1.12]
BMI (kg/m^2^) mean (SD)	23.6 (4.3)	26.5 (7.6)	0.71	1.1 [1.03–1.20]
Ca125 (U/mL) mean (SD)	84.8 (103.4)	93.4 (88.1)	0.73	NS
Previous ovarian surgery, n (%) No Yes			0.44	NS
	64 (77.1%)	19 (22.9%)		
	6 (66.7%)	3 (33.3%)		
Hormonal therapy after procedure, n (%) No Yes			0.22	2.76 [0.88–8.63]
	34 (70.8%)	14 (29.2%)		
	36 (81.8%)	8 (18.2%)		
**US Ovarian & Endometrioma features**				
Cyst size (mm) median [Q1-Q3]	59.5 [50–72]	72.5 [62–77]	0.12	1.03 [1–1.06]
Localization Bilateral Unilateral			0.75	NS
	11 (73.3%)	4 (26.7%)		
	59 (76.6%)	18 (23.4%)		
Cyst septum Not Yes			0.76	NS
	56 (77.8%)	16 (22.2%)		
	13 (72.2%)	5 (27.8%)		
AFC, mean (SD)	10.8 (6.7)	7.6 (6.6)	0.06	NS

AFC = antral follicle count; BMI = body mass index; CI = confidence interval; NS = not significant; HR = hazard ratio; SD = standard deviation; US = ultrasound

Further analysis revealed an increased risk of recurrence with larger cyst size (Fig. 4b), with a sensitivity of 42%, specificity of 33%, positive predictive value of 15%, and negative predictive value of 68% using a 6.5 cm cut-off.

Pain improved or resolved in most patients at six months following either procedure (Fig. 4c). However, a higher proportion of patients in the sclerotherapy group experienced pain improvement or resolution compared with those who underwent laparoscopic surgery (88.9%, 49/57 vs. 65.7%, 21/35), with the difference approaching statistical significance (*p* = 0.05). Cytological and pathological analyses confirmed benign findings in all cases.

## Discussion

This multicentre, randomised clinical trial demonstrates that ultrasound-guided ethanol sclerotherapy is a safe and cost-effective alternative to laparoscopic cystectomy for the treatment of ovarian endometriomas. Sclerotherapy achieved comparable recurrence rates and pain relief, with fewer complications and significantly lower costs. The procedure was performed on an outpatient basis without anaesthesia, offering additional logistical and patient-centred advantages. Although not statistically significant, a trend towards fewer major complications was observed in the sclerotherapy group.

The overall complication rate was low in both treatment groups, in line with previously published data. Importantly, major complications (Clavien-Dindo grade III) were more frequently observed in the laparoscopic surgery group, supporting existing evidence that associates laparoscopy with increased morbidity, particularly in cases complicated by adhesions [8]. Our findings also match a meta-analysis by Kim et al., which reported a major complication rate for sclerotherapy of 1.7% [25].

Recurrence rates for both techniques were within reported ranges (15–30% for surgery [19, 20].; ~ 23% for sclerotherapy [25]), and our findings support the comparable effectiveness of both treatments. Variability in recurrence rates may be partly attributed to differences in sclerotherapy protocols, particularly ethanol retention time. In our study, a 15-min retention period was selected to reduce the risk of ethanol extravasation, which can result in complications such as abdominal pain and adhesions. Previous evidence suggests that retention times shorter than 10 min are associated with increased recurrence rates, whereas extending the retention beyond 20 min does not appear to provide additional benefit [23].

Multivariate analysis did not identify specific risk factors for recurrence, although larger cyst size showed a trend toward significance. Cysts over 6.5 cm in diameter were associated with a higher recurrence risk, consistent with other studies suggesting less favorable outcomes for larger cysts [19, 26]. However, some studies argue that cyst size does not significantly impact recurrence, highlighting the complexity of predicting recurrence [27, 28]. Hormonal treatments did not prevent recurrence in our study, consistent with a recent meta-analysis showing no significant reduction in recurrence with hormonal therapy compared to expectant management [29]. However, long-term use of oral contraceptives or progestins has been linked to reduced recurrence risk. Further studies with longer follow-up are needed to assess the role of hormonal therapy in preventing recurrence after sclerotherapy or surgery.

Sclerotherapy’s cost-effectiveness was evident, with direct hospital costs being nearly five times lower than those of laparoscopic surgery—an important consideration in healthcare resource allocation.

Interestingly, although patients with symptomatic extraovarian endometriosis—commonly associated with endometriomas [30]—were excluded from enrollment, a substantial proportion of patients in the surgical group were found to have these lesions and received tailored treatment at the discretion of the surgeon. Despite this potential advantage for the surgical arm, the sclerotherapy group achieved not only comparable but actually higher rates of pain improvement or resolution at six months, with the difference approaching statistical significance. Given that laparoscopic surgery itself may be associated with postoperative pain, these findings suggest that ultrasound-guided sclerotherapy, as a minimally invasive approach, could offer superior pain control in selected patients. Prior studies have similarly reported complete pain resolution in up to 48% of sclerotherapy case [31], supporting its role as a minimally invasive and effective pain management strategy. Furthermore, approximately 10% of patients were asymptomatic at baseline, likely due to prior hormonal treatment, which may have influenced post-treatment outcomes. These findings underscore the importance of hormonal therapy in the management of endometriomas and highlight the need for further research into its optimal use alongside minimally invasive approaches.

### Strengths and limitations

A major strength of this study lies in its randomised design and multicentre participation, which enhances the generalisability and external validity of the findings. The prospective collection of data on complications, recurrence, and pain also allowed for robust safety and efficacy assessment. Nevertheless, the trial was terminated early following an interim analysis conducted at midpoint enrollment, which assessed safety and recurrence. Although no major safety concerns were confirmed, the declining recruitment rate and the limited likelihood of reaching the planned sample size led to early discontinuation due to futility. Additionally, some patients declined the assigned treatment arm, further reducing the per-protocol population. Although surgical technique varied slightly between participating centres, potentially introducing a degree of heterogeneity, this variability reflects the realities of clinical practice across different hospital settings. Despite this, the prospective, multicentre design of our study enhances its external validity. The consistent collection and reporting of complication data across sites strengthens the evidence supporting the safety and feasibility of ultrasound-guided sclerotherapy as a minimally invasive treatment option for endometriomas. Finally, although the current analysis does not focus on ovarian reserve or fertility outcomes, a separate publication is planned to evaluate the specific impact of both techniques on AMH levels, antral follicle count (AFC), and subsequent fertility, to improve clarity and avoid dilution of key findings.

In conclusion, US-guided ethanol sclerotherapy is a promising alternative to laparoscopic surgery for treating endometriomas, offering low complication rates and similar recurrence outcomes. It is also a cost-effective option, particularly in outpatient settings. However, further research with longer follow-up is needed to assess its long-term efficacy, especially when combined with hormonal therapy.

## Data Availability

See https://data.mendeley.com/datasets/7d5m2g9bcy/1.
